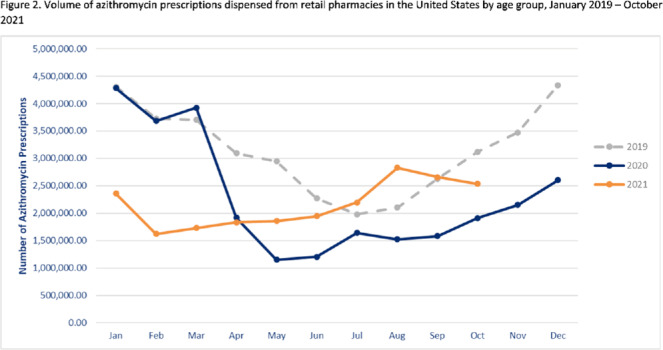# Outpatient antibiotic prescribing during the COVID-19 pandemic–United States, January 2019–October 2021

**DOI:** 10.1017/ash.2022.59

**Published:** 2022-05-16

**Authors:** Destani Bizune, Sharon Tsay, Sarah Kabbani, Lauri Hicks

## Abstract

**Background:** Improving antibiotic use is a key strategy to combat antimicrobial resistance. Here, we have described national outpatient antibiotic prescribing trends during the COVID-19 pandemic. We compared the monthly numbers of prescriptions in 2020–2021 to those from 2019 to describe the impact of the pandemic and to highlight areas for improvement. **Methods:** We used the IQVIA National Prescription Audit (NPA) data set to identify all antibiotic prescriptions dispensed from US retail pharmacies during January 2019–October 2021. We calculated the percentage change in volume of prescriptions for each month during the pandemic (beginning in March 2020) compared to the baseline (defined as the corresponding month in 2019). Data were characterized by patient age group (0–19 years, 20–64 years, ≥65 years) and antibiotic class and drug, including azithromycin. **Results:** Antibiotic prescriptions were lower than baseline during March 2020–June 2021. The greatest decrease in antibiotic prescribing volume occurred in May 2020 (40.0% lower than May 2019) (Fig. 1), with the greatest decreases among children 0–19 years of age. However, prescribing was similar to baseline levels in July–August 2021 (Fig. 1). Specifically, azithromycin prescribing exceeded the 2019 baseline by 11.0% in July and further to a 34.5% increase in August 2021 (Fig. 2). Increases in azithromycin prescribing in August 2021 were observed across all age groups: 20–64 years (46.9% above baseline), ≥65 years (25.3% above baseline), and children 0–19 years (7.8% above baseline). **Conclusions:** Antibiotic prescribing volume was lower during 2020 and the first half of 2021 compared to the corresponding months in 2019. Decreases in outpatient antibiotic prescriptions during the pandemic likely reflect decreased utilization of outpatient healthcare and decreased transmission of non–COVID-19 infections secondary to nonpharmaceutical interventions (eg, masking, social distancing, school closures). However, outpatient antibiotic prescribing levels in general, and azithromycin prescribing in particular, approached or exceeded prepandemic levels in July and August 2021. Ongoing surveillance and sustained outpatient antibiotic stewardship efforts are needed to optimize antibiotic use during the COVID-19 pandemic and beyond.

**Funding:** None

**Disclosures:** None

**Figure:**
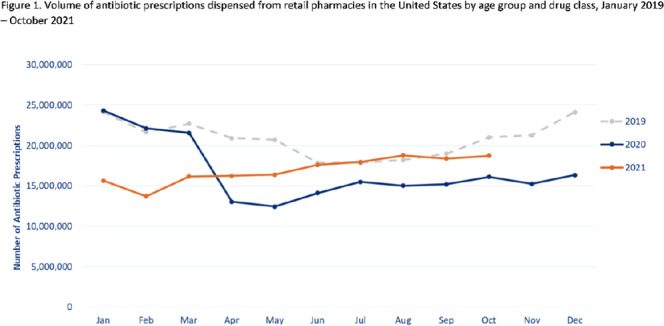

**Figure:**